# Neuromorphological Alterations in the Somatosensory System of Adolescent Idiopathic Scoliosis: A Systematic Review of Magnetic Resonance Imaging Studies

**DOI:** 10.3390/children13040499

**Published:** 2026-04-01

**Authors:** Qikai Wu, Zhengquan Chen, Kang Chen, Xin Li, Haibin Guo, Xiangyue Zhou, Juping Liang, Qing Du

**Affiliations:** 1 Department of Rehabilitation, Xinhua Hospital, School of Medicine, Shanghai Jiaotong University, Shanghai 200092, China; wuqikai5723@xinhuamed.com.cn (Q.W.); jsckly@sjtu.edu.cn (K.C.); lixin02@xinhuamed.com.cn (X.L.); guohaibin@xinhuamed.com.cn (H.G.); xkirei1999@sjtu.edu.cn (X.Z.); 2 Yuanshen Rehabilitation Institute, Shanghai University of Medicine & Health Sciences, Shanghai 201318, China; 3 Shanghai Yangpu District Mental Health Center, Shanghai 200093, China; zhengquan.chen@rmit.edu.au; 4 School of Health and Biomedical Sciences, Royal Melburne Institute of Technology University, Bundoora, VIC 3083, Australia; 5 Department of Rehabilitation Medicine, Shanghai Jiao Tong University Affiliated Sixth People’s Hospital, 600 Yishan Rd, Shanghai 200233, China

**Keywords:** adolescent idiopathic scoliosis, somatosensory system, neuroimaging, cerebellum, vestibular system

## Abstract

Background/Objectives: This systematic review synthesizes MRI evidence to characterize neuromorphological alterations in somatosensory and vestibular brain regions among adolescents with idiopathic scoliosis (AIS). Methods: This systematic review was conducted in accordance with the PRISMA 2020 guidelines. We systematically searched five databases from inception to January 2026 for case–control MRI studies comparing AIS patients (10–18 years) with healthy controls. Two reviewers independently screened studies, extracted data, and assessed the risk of bias using the Newcastle–Ottawa Scale. Results: Across 15 studies (1270 participants), AIS patients demonstrated consistent neuromorphological alterations: (1) lower cerebellar tonsil position (0.9–2.8 mm below baseline), with ectopia incidence correlating with curve severity; (2) cortical thickening in bilateral medial regions but thinning in left paracentral areas; (3) left-dominant white matter volume increases and impaired microstructure in the corpus callosum; and (4) left-sided vestibular morphological changes, including a more vertical semicircular canal. Conclusions: AIS is associated with consistent neuromorphological alterations in key somatosensory and vestibular regions, supporting a potential neuroanatomical basis for impaired sensorimotor integration in its pathogenesis. It should be noted that substantial heterogeneity among the included studies prevented a meta-analysis, and the cross-sectional design limits causal interpretations Registration: This systematic review was registered in PROSPERO (CRD42024577195).

## 1. Introduction

Adolescent idiopathic scoliosis (AIS) is a three-dimensional spinal deformity affecting children aged 10–18 years, and is typically accompanied by sensorimotor dysfunction [1]. This condition affects approximately 1–3% of adolescents in the at-risk population [2], with a marked female predominance and higher prevalence among those over 15 years of age [3]. The sensorimotor deficits observed in AIS patients include impaired postural control ability, characterized by increased postural sway and reduced stability during quiet standing, particularly when visual or proprioceptive inputs are altered [4,5]. Balance deficits are also consistently reported, with patients showing poorer performance on both static and dynamic balance tasks compared with healthy controls [6]. Proprioceptive dysfunction manifests as an impaired joint position sense, most commonly at the ankle and spine [7,8]. The somatosensory system is critical for maintaining spinal alignment and postural control. Impairment at any level of the somatosensory system, particularly within central integration pathways, may exacerbate spinal deformity, and in turn, spinal deformity may further compromise the accuracy of somatosensory feedback [9]. Neurophysiological studies consistently report abnormal somatosensory-evoked potential in a substantial proportion of AIS patients (up to 14.3%) [10], suggesting that central processing abnormalities may underlie these peripheral deficits. The presence of delayed or attenuated cortical responses following peripheral nerve stimulation points to altered transmission or processing of somatosensory information at the spinal or supraspinal level [10]. Identifying the structural correlates of these functional disturbances could inform targeted rehabilitation strategies focusing on proprioceptive and balance training, providing strong translational justification for a focused synthesis of somatosensory system alterations in AIS. Magnetic Resonance Imaging (MRI) studies have revealed alterations in gray matter morphology in AIS patients, white matter microstructural integrity, and functional connectivity patterns within these regions compared with healthy controls [11], with some studies linking these structural and functional changes to clinical measures of balance and proprioceptive performance [11]. These findings suggest the involvement of central nervous system structures involved in sensory integration and motor control. However, despite accumulating evidence, the precise anatomical location and brain functional network alterations in AIS remain far from established [11,12]. Specifically, there is poor spatial concordance across studies regarding the specific brain regions affected, with findings ranging from cortical thickening in some areas to thinning in others, and from white matter increases in certain tracts to microstructural disruption elsewhere [11,13,14]. Additionally, the relationship between neuroimaging findings and clinically meaningful parameters, such as curve severity or postural control deficits, remains weakly characterized [12,15]. This lack of clarity not only impedes mechanistic understanding but also precludes the development of neuroimaging-based biomarkers for clinical application [11,12].

MRI enables the non-invasive assessment of brain structure and functional network dynamics, yet its application in AIS research remains underutilized. This systematic review addresses these gaps through: (1) synthesizing the existing evidence under rigorous methodological quality assessment; (2) integrating structural and functional neuroimaging data across modalities; and (3) critically appraising methodological limitations to guide future large-scale, multimodal longitudinal studies.

## 2. Materials and Methods

### 2.1. Study Protocol and Registration

This systematic review was registered with PROSPERO (CRD42024577195) and follows the Preferred Reporting Items for Systematic Reviews and Meta-Analyses (PRISMA) 2020 statement (see Supplementary Materials, PRISMA_2020_checklist) [16] for reporting and the Cochrane Handbook for Systematic Reviews of Interventions (version 5.1.0) [17] for methodology.

### 2.2. Search Strategy

To identify relevant studies from inception to January 2026, we systematically searched five electronic databases: PubMed (including MeSH terms), the Cochrane Library, Embase (including Emtree terms), Web of Science, and EBSCO (MEDLINE). The search strategy combined keywords and controlled vocabulary terms related to three core concepts: (1) the population (“adolescent idiopathic scoliosis”, “AIS”, “scoliosis”); (2) the imaging modality (“neuroimaging”, “magnetic resonance imaging”, “MRI”, “diffusion tensor imaging”, “DTI”, “voxel-based morphometry”, “VBM”, “functional MRI”, “fMRI”); and (3) the system of interest (“somatosensory”, “sensorimotor”, “brain”, “cerebrum”, “cerebellum”, “cortical”, “white matter”). Boolean operators (AND, OR) were used to combine terms within and across these concepts. The search was restricted to human studies published in English prior to January 2026. Where applicable, database filters for age (adolescent: 13–18 years) and publication type were applied. The full search strategy (see Supplementary Materials, Search strategy) has been presented in the Supplemental Materials. Additionally, the reference lists of relevant review articles were manually searched and screened for additional eligible studies.

### 2.3. Eligibility Criteria

Studies were eligible if they: (1) used a case–control design involving adolescents with AIS (aged 10–18 years) and age/sex-matched healthy controls; (2) employed MRI (structural, diffusion, or functional) to investigate brain or vestibular characteristics; and (3) had a stated objective to examine neural mechanisms related to somatosensory processing, sensorimotor integration, or sensorimotor control in AIS. This purpose-driven approach was supplemented by anatomical reference standards (Brodmann’s Areas), with somatosensory-related regions defined as including but not limited to the somatosensory cortex, visual cortex, vestibular-related cortices, thalamus, brainstem sensory nuclei, or associated white matter pathways. A structured eligibility criteria table is provided in Table 1.

Studies were excluded if they: (1) included participants with non-idiopathic scoliosis or other major neurological conditions; (2) did not focus on somatosensory processing, sensorimotor integration, or sensorimotor control as a primary research objective; (3) lacked a healthy control group; (4) were not original research (reviews, case reports, conference abstracts, editorials); or (5) had no full text available.

### 2.4. Study Selection

**Figure 1:**
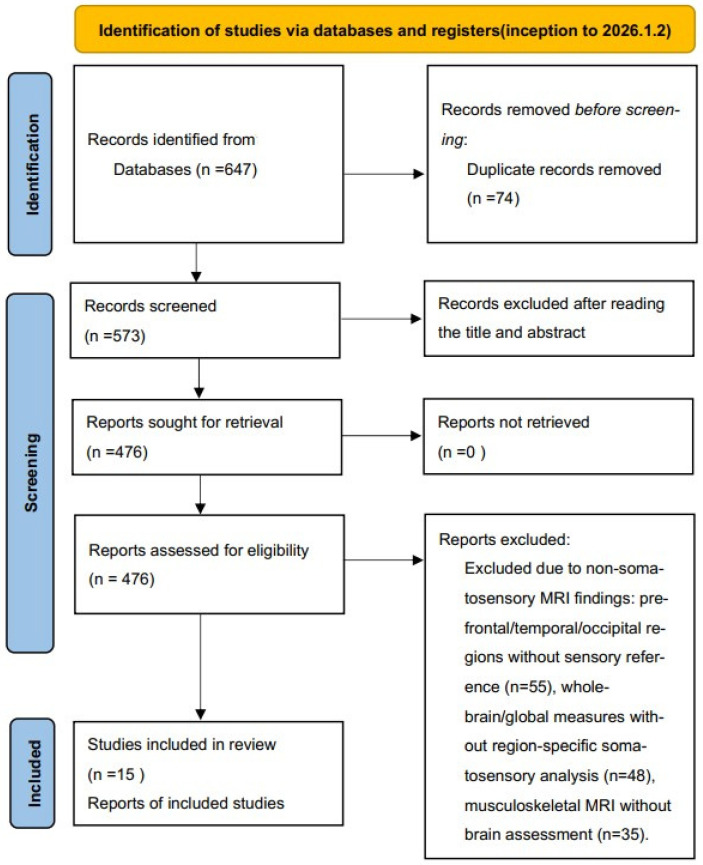
Study selection flowchart.

The identified records from all databases were combined and de-duplicated using End-note X9 software. Two reviewers independently conducted the screening, first based on titles and abstracts, and subsequently by reviewing the full texts of potentially eligible studies. Any disagreement was resolved by two researchers through negotiation and discussion until a consensus was reached. If a consensus could not be reached, a third reviewer was consulted to make the final decision. The systematic search yielded a total of 647 records across the five databases. After the removal of 74 duplicate records, 573 records remained for screening. The remaining 476 reports were sought for retrieval and assessed via full-text review. Of these, 461 reports were excluded for the following reasons: Not AIS patients (n = 151). Control group is not healthy people (n = 172). MRI target is not somatosensory system (n = 138). A total of 15 studies met all eligibility criteria and were included in this systematic review. The screening process is illustrated in the PRISMA flow diagram (Figure 1). For the purpose of this systematic review, the somatosensory system was operationally defined to include the postcentral gyrus (primary somatosensory cortex) as the principal cortical region, alongside key subcortical structures—specifically, the thalamus (with emphasis on the ventral posterior nuclear complex) and the brainstem (encompassing nuclei involved in afferent sensory processing)—as well as the white matter pathways that interconnect these components, including the superior thalamic radiation and the medial lemniscus. Accordingly, studies were excluded if they reported MRI-based findings exclusively in brain regions falling outside this predefined somatosensory circuitry—such as the prefrontal, temporal, or occipital lobes; basal ganglia; cerebellar hemispheres; insula; or white matter tracts unrelated to sensory transmission.

### 2.5. Data Extraction

Two reviewers independently completed the information extraction for the included studies. A standardized data extraction form was developed prior to the review, which included: publication year, author name, number of participants, age of participants, type of scoliosis, Cobb angle of patients with AIS, and outcome measures. MRI-related details extracted from the included studies included: level of adjustment, device, MR imaging scanner, sequence, parameter, and imaging targets. In case of any disagreement, a third reviewer was consulted for adjudication.

### 2.6. Quality Assessment

The methodological quality and risk of bias of the included case–control studies were assessed independently by two reviewers using the Newcastle–Ottawa Scale (NOS) [18]. NOS is based on (1) clarity of the study objective, (2) sample selection (representativeness of the sample; sample size; response rate; ascertainment of exposure), (3) comparability (control of confounding factors; comparability of participants from different outcome groups), (4) outcome (assessment; statistical tests). Each reviewer assigned a score based on the NOS star system. Disagreements in scoring were resolved by discussion, or by consultation with a third reviewer if needed.

### 2.7. Data Synthesis

Due to substantial heterogeneity in imaging methodologies, including variations in MRI sequences, brain regions examined, and analytical approaches, quantitative data synthesis (meta-analysis) was not performed. For qualitative synthesis, findings were organized thematically according to key anatomical components of the somatosensory system. Within each domain, comparison matrices were used to evaluate the consistency, direction, and strength of evidence across studies. This approach allowed us to identify patterns of convergence or divergence in reported neuromorphological alterations.

## 3. Results

### 3.1. Study Results

We obtained 647 relevant studies from five databases, of which 74 studies were removed as duplicates. The remaining 73 studies were evaluated based on relevance and publication type, resulting in the exclusion of 476 articles with obviously irrelevant topics or non-control studies. After full-text reading, 15 case–control studies that met the eligibility criteria were included in this systematic review [10,12,13,15,19,20,21,22,23,24,25,26,27,28]. The screening flow diagram is illustrated in Figure 1.

### 3.2. Study Characteristics

The details of the included studies are presented in Table 2. The MRI-related details of the included studies are summarized in Table 3. This review encompasses studies published from 1999 to 2022, including a total of 1270 participants: 822 patients with AIS and 448 healthy control subjects in the control groups. All included studies were case–control studies published in English. The sample sizes in most of the included studies were small, with 10 studies involving fewer than 100 participants in total. The mean age of patients with AIS ranged from 13.6 to 15.7 years across studies (median: 14.8 years), with individual ages spanning from 10 to 20 years in studies that reported age ranges. Control groups were generally well-matched for age, with mean ages ranging from 12.3 to 18.7 years. The majority of curves were right thoracic (most common), though right lumbar, thoracolumbar, and left thoracic curves were also reported. Each MRI protocol was used in different parts of the brain. For example, diffusion tensor imaging (DTI) was used for white matter integrity, high-resolution T1-weighted imaging for volumetry and cortical thickness, T2-weighted or CSF flow-sensitive sequences for cerebellar and foramen magnum evaluation and vestibular MRI protocols assessing labyrinthine morphology.

### 3.3. Differences in Somatosensory Systems Related to Neuromorphology Comparing AIS Patients to Controls

#### 3.3.1. Cerebellar Tonsillar Level

Five studies investigated the cerebellar tonsil position in AIS patients using the basion–opisthion (BO) line as reference [10,19,20,21,29]. Tonsillar ectopia, defined as the tonsillar descent below the BO line, was observed in both upright and supine positions, with a greater descent in the upright position [20]. Patients with AIS exhibited tonsil levels 0.9–2.8 mm below the BO line, with ectopia incidence ranging 34.5–48.0% [19,20,21]. The highest rates occurred in double thoracic curvature (62.5%) [20] and syringomyelia (66.7%) patients [30]. Ectopia incidence correlated with scoliosis severity: Chau et al. reported 5.9% (Cobb 10–19°), 6.7% (20–39°), and 27.3% (>40°) [29], while Sun et al. found 35.3% (40–59°), 36.6% (60–89°), and 16.6% (>90°) [20]. Low-lying cerebellar tonsils (≥2 mm below the BO line) were observed in 34.5–48.0% of patients with AIS, with the highest incidence in those with double thoracic curvature (62.5%) and Cobb angles >40° (27.3%).

#### 3.3.2. Cerebral Cortex

Two studies investigated structural and functional differences in the cerebral cortex between AIS patients and healthy participants [12,15]. Regarding structural morphology, one study reported that, compared to healthy subjects, AIS patients exhibited a thicker cortex in the medial part of both hemispheres (with significant thickening in the right paracentral and left superior-frontal regions) alongside a thinner cortex in specific left-hemispheric areas, including the Sylvian fissure, precentral, and lateral occipital regions [12]. In terms of functional activation, another study using functional MRI found that AIS patients showed significant hyperactivation of the supplementary motor area, a region that receives intensive sensory input from the primary somatosensory cortex [15]. Compared with controls, patients with AIS demonstrated regional cortical thickening (right paracentral, left superior-frontal) and thinning (left Sylvian fissure, precentral, lateral occipital), as well as hyperactivation of the supplementary motor area on fMRI.

#### 3.3.3. White Matter

Five studies investigated white matter alterations in AIS, with one assessing volumetric changes [13] and four focusing on corpus callosum microstructure and volume [22,23,24,25]. AIS patients exhibited significantly larger normalized white matter volumes in multiple left-hemispheric regions, including the frontal, parietal, and temporal lobes, as well as the thalamus [13]. Diffusion tensor imaging revealed reduced fractional anisotropy (FA) in the genu and splenium of the corpus callosum, including fibers connecting the somatosensory and visual cortices [23]. Kong et al. further reported decreased FA and increased mean diffusivity in the medulla oblongata and cervical spinal cord (C1–C5) [25]. In contrast, increased FA was observed in fibers linking motor areas to the cingulate gyrus [22]. Volume-based morphometry confirmed white matter attenuation in the genu and left internal capsule in left thoracic AIS [24], suggesting these microstructural alterations may contribute to somatosensory dysfunction. White matter alterations in patients with AIS included: (1) increased normalized volume in left frontal, parietal, temporal, and thalamic regions; (2) reduced FA in corpus callosum genu and splenium, medulla oblongata, and cervical spinal cord; (3) increased FA in cingulate motor fibers; and (4) reduced volume in corpus callosum genu and left internal capsule among patients with left thoracic curves.

#### 3.3.4. Vestibular System

Three studies examined vestibular system morphology in AIS [26,27,28]. Neuromorphological alterations were predominantly left-sided, including a more vertical and laterally positioned left semicircular canal [26], reduced inter-canal distances and angles [28], and larger canal angles [27]. Right-sided differences were limited to larger semicircular canal angles [27]. Volumetric analyses revealed no significant asymmetries in overall vestibular or semicircular canal volume between AIS patients and controls, or between hemispheres [28]. Notably, thoracolumbar curves exhibited smaller inter-canal angles and greater lateral semicircular canal asymmetry compared to lumbar curves [26]. These results indicate that the vestibular system in AIS patients, particularly the left semicircular canal, exhibits specific geometric remodeling and spatial orientation abnormalities. This alteration demonstrates a left-sided predominance, is associated with thoracolumbar curve types, and occurs without accompanying changes in overall volume.

### 3.4. Quality Appraisal

The risk of bias in the 15 included case–control studies was assessed using the Newcastle–Ottawa Scale (Table 4). Overall, the evidence was rated as having a medium risk of bias. Although all studies provided adequate case definitions and reported no non-response, limitations were noted regarding the representativeness of cases and the selection of controls. Comparability was satisfactory except in one study. Specific concerns about selection bias included incomplete control selection in three studies and inadequate representation of the exposure group in five studies. However, considerable methodological heterogeneity was present across the studies, primarily due to variations in MRI sequences and measurement methods. The insufficient reporting of demographic data, together with these methodological inconsistencies, contributed to an elevated risk of bias in the overall evidence.

## 4. Discussion

This systematic review demonstrated a multi-level dysfunction within the somatosensory neural network in AIS, spanning from the vestibular system and cerebellum to cerebral cortical and white matter pathways. The structural alterations identified may reflect underlying neurodevelopmental anomalies [31] although a contribution from secondary remodeling due to long-term abnormal biomechanical loading cannot be ruled out [32]. Specifically, AIS patients showed inferior displacement of the cerebellar tonsils, with a higher prevalence of tonsillar ectopia that correlates with scoliosis severity. Differences were also observed in cortical thickness, regional brain volume, and patterns of cortical activation. Furthermore, alterations in white matter volume and microstructural integrity highlighted disrupted neural connectivity. Notably, morphological deviations in the vestibular system display a left-sided predominance [30], suggesting a potential lateralized influence on sensorimotor integration [33]. These findings point to widespread neuromorphological disturbances that may underpin the postural and sensory integration deficits observed in AIS [15].

Our findings align with the broader understanding that somatosensory dysfunction contributes to AIS pathogenesis [34], yet they delineate a more specific neuroanatomical framework. One prior systematic review highlighted impaired standing balance in AIS, integrating evidence from diverse methodologies (e.g., posturography, motion capture) [35]. In contrast, this review concentrates on synthesizing morphometric MRI evidence to characterize the underlying structural substrates of such functional deficits. By exclusively focusing on case–control studies with healthy comparisons and MRI-based morphometry, our work provides a focused analysis of structural alterations within the somatosensory network.

The synthesized evidence reveals a consistent pattern of alterations across key levels of this network [36]. At the subcortical level, AIS is associated with a higher prevalence of cerebellar tonsillar ectopia [37], the severity of which correlates with the Cobb angle, suggesting a possible role of craniocervical junction anatomy in sensorimotor integration [37]. Regarding cortical structure, patients exhibit regionally specific changes in gray matter thickness and activation patterns, particularly in areas involved in sensory processing and motor planning [34,38]. Furthermore, white matter microstructural alterations, especially within the corpus callosum and left hemispheric pathways, impair efficient interhemispheric communication and disrupt long-range networks essential for sensorimotor function. For instance, these changes likely hinder the transfer of sensory information into coordinated motor commands across hemispheres [38].

In summary, these multi-level MRI-detectable differences support the view that AIS may involve a widespread disruption of the somatosensory neural architecture [35,38]. While previous work established the functional consequence of balance impairment [39], this review delineates its potential structural correlates. The left-lateralized predominance of many findings suggests a plausible, yet speculative, link to the typical rightward curvature of the spine, warranting verification in future lateralization-specific studies [36]. Thus, our synthesis not only confirms the importance of the somatosensory system in AIS but also maps its specific neuromorphological signature, offering targets for future research into mechanisms and imaging biomarkers.

The evidence synthesized in this review challenges the view of AIS as purely a skeletal deformity, highlighting instead its strong associations with neurological alterations in the somatosensory system and vestibular system. Future efforts in early diagnosis should integrate neuroimaging biomarkers—such as cerebellar tonsillar position, cortical thickness, and corpus callosum fractional anisotropy (FA) values. Concurrently, rehabilitation strategies must shift from a purely “spinal orthopedic” approach toward a “brain–spine” co-modulation model. By employing targeted sensory stimulation and task-specific training aimed at sensorimotor circuits, it may be possible to maximize neuroplasticity, thereby improving postural control and potentially mitigating curve progression.

Regarding the generalizability of our findings, the included studies predominantly enrolled female adolescents with right thoracic curves, though some studies include males and patients with primary lumbar or left thoracic curves [13]. The geographic distribution was primarily concentrated in East Asian [11,39] and European populations [15,40]. These factors suggest that the samples demonstrate acceptable generalizability across sex, ethnicity, and AIS subtypes. However, ten of the 15 included studies had sample sizes below 100 participants, suggesting that the observed neuromorphological patterns require validation in larger cohorts before broad generalization can be assumed. Despite these constraints, the consistent alterations across the cerebellar tonsillar position [11], cortical asymmetries [12,13], and white matter microstructure [14] offer preliminary candidate imaging biomarkers for early AIS identification and targeted sensorimotor interventions. Future efforts to stratify sensorimotor deficit risk and develop precision interventions will require prospective multicenter studies with diverse patient representation.

This systematic review has several limitations that should be considered when interpreting the findings. First, the evidence is predominantly derived from cross-sectional studies, which precludes any determination of causality. It therefore remains unclear whether the identified neuromorphological alterations precede and contribute to the development of AIS or are secondary consequences of the spinal deformity and its associated abnormal biomechanics. Second, significant methodological heterogeneity was observed across studies in MRI acquisition protocols and analytical techniques, limiting the comparability and synthesis of findings. Third, the inconsistent reporting of demographic and clinical confounders (e.g., Cobb angle subtypes, bracing status) in many studies hampers the ability to adjust for potential confounding factors. These limitations highlight the need for future large-scale, longitudinal studies employing standardized neuroimaging protocols to determine the temporal sequence of these neuromorphological changes, clarify their etiological role, and validate their potential as neuroimaging biomarkers for AIS. Fourth, the restriction to English-language publications represents a potential source of language bias. While the majority of neuroimaging research in AIS is published in English, and a preliminary review suggested that non-English studies meeting our eligibility criteria were limited, we cannot exclude the possibility that relevant findings published in other languages were missed. Future reviews may consider including non-English studies with professional translation services to further enhance the comprehensiveness and generalizability of findings.

## 5. Conclusions

This systematic review synthesizes consistent evidence for multi-level neuromorphological alterations in the somatosensory and vestibular systems of AIS patients. These findings point to a neuroanatomical basis for sensorimotor dysfunction and offer candidate imaging biomarkers that could inform early detection or targeted interventions. However, causality remains unclear due to cross-sectional designs, small samples, and limited generalizability. Future research requires large-scale longitudinal studies with standardized protocols to validate these biomarkers and establish their clinical utility.

## Figures and Tables

**Table 1:** Eligibility criteria of studies.

Category	Inclusion Criteria	Exclusion Criteria
Study Design	Case–control studies	Non-case–control designs (e.g., reviews, case reports, animal studies)
Participants	Adolescents aged 10–18 years	Participants outside the 10–18 age range
Exposure/Condition	Clinical diagnosis of Adolescent Idiopathic Scoliosis	Non-idiopathic scoliosis (e.g., congenital, neuromuscular) or comorbid major neurological/developmental disorders
Control Group	Healthy controls, matched to cases for age and sex	Unhealthy controls; controls not matched for age and/or sex
Outcome and Measures	MRI-based assessment of neuromorphology in brain or vestibular structures linked to somatosensory function	Studies not using MRI, or MRI not targeting somatosensory-related brain/vestibular morphology

**Table 2:** Characteristics of included studies.

Author, Year	No. of Participants (% Male)	Age (y), Median/ Mean (SD)	Type of Curve	Cobb Angle (°), Median/ Mean (SD)	Outcome Measures
Noriega et al., 2022 [22]	AIS: 22 (22.7) Control: 18 (55.5)	AIS: 14.73 (3.03) Control: 12.33 (2.43)	not reported	not reported	FA (fractional anisotropy) the number of streamlines
Carry et al., 2020 [27]	AIS: 20 (0) Control: 19 (0)	AIS: 14.6 (1.9) Control: 22.0 (7.8)	right thoracic (n = 10) right lumbar (n = 2) biphasic * (n = 8)	right thoracic: 48.4 (10.0) right lumbar: 55.0 (7.1) biphasic: 46.1 (10.2)	the morphoanatomy of vestibular system
Xue et al., 2018 [23]	AIS: 69 (0) Control: 40 (0)	AIS: 14.5 (2.2) Control: 14.6 (1.04)	right thoracic (n = 65) right thoracolumbar (n = 4)	33.55 (13.9)	1. microstructural changes in the corpus callosum 2. microstructural changes in interhemispheric white matter fiber tracts interconnecting both cerebral hemispheres
Chau et al., 2016 [29]	AIS: 91 (0) Control: 49 (0)	AIS: NA Control: 14.6 (NA)	right thoracic (n = 91)	severe: 56.4 (11.6) moderate: 25.8 (11.1) mild: 14.3 (5.0)	1. somatosensory-evoked potential 2. cerebellar tonsillar level
Hitier et al., 2015 [26]	AIS: 17 (23.5) Control: 9 (33.3)	AIS: 15.47 (1.84) Control: 16.7 (1.50)	thoracic (n = 8) lumbar (n = 5) thoracolumbar (n = 4)	26.7 (8.3)	1. orientation of the lateral semicircular canal in the parasagittal and the frontal plane 2. position of the three semicircular canals 3. morphologic asymmetry between the right and left vestibule 4. function of the lateral semicircular canal
Lee et al., 2015 [21]	AIS: 25 (0) Control: 18 (0)	AIS: 14.9 (2.3) Control: 18.7 (4.5)	thoracic (n = 9) lumbar (n = 2) thoracolumbar (n = 14)	Range (15–63) mean 26.3 ± 11.4	cerebellar tonsillar position
Kong et al., 2014 [25]	AIS: 13 (0) Control: 13 (0)	AIS: 13.6 (11–16) Control: 13.9 (12–15)	right thoracic (n = 13)	24.7 (16–37)	mean values of fractional anisotropy mean diffusivity
Wang et al., 2012 [14]	AIS: 50 (0) Control: 40 (0)	AIS: 14.46 (12–17) Control: 14.53 (12–17)	right thoracic (n = 50)	48.7 (20–90)	thickness of cerebral cortex
Shi et al., 2010 [28]	AIS: 20 (0) Control: 20 (0)	AIS: 14.7 (1.8) Control: 15.1 (2.4)	right thoracic (n = 20)	32.6 (19.1)	the morphoanatomy of vestibular system
Domenech et al., 2011 [15]	AIS: 10 (20) Control: 10 (30)	AIS: 15.2 (14–16) Control: 14.7 (14–16)	right thoracic (n = 6) right thoracolumbar (n = 2) left thoracic (n = 1) left lumbar (n = 1)	35 (27–55)	cortical activation
Shi et al., 2009 [24]	AIS: 29 (0) Control: 28 (0)	AIS with left thoracic curve: 14 (11–16) AIS with right thoracic curve: 15 (13–17) Control: NA	left thoracic (n = 9) right thoracic (n = 20)	Not reported	corpus callosum volume
Liu et al., 2008 [13]	AIS: 20 (0) Control: 26 (0)	AIS: 14.1 (11–18) Control:14.8 (11–18)	right thoracic (n = 20)	53 (37–68)	volumes of 99 preselected neuroanatomical regions
Sun et al., 2007 [20]	AIS: 203 (13.3) Control: 86 (50)	AIS: 15.7 (1.8) Control: 14.6 (1.6)	not reported	>40	cerebellar tonsillar level
Chu et al., 2007 [19]	AIS: 69 (0) Control: 36 (0)	15 (11–18)	thoracic curve (n = 40) lumbar curve (n = 27) thoracolumbar curve (n = 2)	36 (26–52)	1. cerebellar tonsillar level 2. the anteroposterior diameter and area of the foramen magnum 3. the peak velocity of cerebrospinal fluid flow through the foramen magnum 4. somatosensory-evoked potential
Cheng et al., 1999 [10]	AIS: 164 (13.4) Control: 36 (58.3)	AIS: group II 14.2 (10–20) Group III 13.6 (10–20) Control: group I 12	thoracic curve (n = 40) lumbar curve (n = 27) thoracolumbar curve (n = 2)	group II (10–45) group III (45–105)	results of abnormal findings tonsillar ectopia syringomyelia somatosensory-evoked potential

* Biphasic: right thoracic/left thoracolumbar curves of similar magnitude.

**Table 3:** MRI-related details of the included studies.

Author, Year	Level of Adjustment	Device	MR Imaging Scanner, Sequence, Parameter	Imaging Targets
Noriega et al., 2022 [22]	age-matched	Achieva 3T MRI; Philips Healthcare, Best, the Netherlands	- T1 anatomical acquisition voxel size of 0.865 × 0.875 × 1 mm; 320 × 320 matrix with 170 sagittal slices - first diffusion acquisition; 32 gradient directions, b = 1000 s/mm^2^, a voxel size of 1.66 × 1.66 × 2 mm; 144 × 144 matrix; 140 axial slices - second diffusion acquisition; b = 1000 s/mm^2^; a voxel size of 1.7 × 1.7 × 2 mm; 128 × 128 matrix with 24 coronal slices	cervical area and whole brain
Carry et al., 2020 [27]	gender-matched	Siemens	- 1.5T - T2-weighted Avonto true fast imaging with steady-state precession sequence - TR: 4.93 ms; TE: 2.16 ms; flip angle: 65°; FOV: 75; slice thickness: 1 mm; matrix: 256 × 192; NEX: 1	vestibular system (semicircular canals)
Xue et al., 2018 [23]	age- and gender-matched	Achieva TX series; Philip Healthcare, Best, the Netherlands	*DTI:* - 3T - single-shot echo-planar imaging sequence - TR: 8667 ms; TE: 60 ms; FOV: 224 × 224 mm^2^; flip angle: 90°; section thickness: 2 mm, no gap; matrix: 112 × 109; NEX: 1, sections: 70; in-plane image resolution: 2 × 2 mm *MRI:* - 3T - T1-weighted 3D fast-field echo imaging sequence - TR: 18 ms; TE: 2.4 ms; FOV: 210 × 210 mm^2^; flip angle: 30°; matrix: 232 × 232; NEX: 1; sections: 200.	Corpus callosum, interhemispheric white matter fiber tracts interconnecting both cerebral hemispheres
Chau et al., 2016 [29]	age- and gender-matched	Gyroscan ASC NT; Philips Medical System, Best, the Netherlands	- 1.5T - turbo spin–echo T1- and T2-weighted sequence - TR: 3.5 s; TE: 120 ms; FOV: 25–40 cm; slice thickness: 4 mm; matrix: 256 × 256	cerebellar tonsillar
Hitier et al., 2015 [26]	school environment-matched	General Electric	- 1.5T - T2 3D fast spin echo sequence - TR: 3500 ms; TE: 110 ms; FOV: 180 × 180 mm^2^; slice thickness: 0.6 mm; matrix: 288 × 288	vestibular system (semicircular canal)
Lee et al., 2015 [21]	Age-gender-matched	G-scan; Esaote SpA, Genoa, Italy	- 0.25T - T2-weighted turbo spin-echo - TE = 120 ms; TR = 3220 ms; slice thickness 4 mm, gap 0.4 mm, field of view 310 × 310 mm, resolution 512 × 512 mm	cerebellar tonsillar
Kong et al., 2014 [25]	Age-gender-matched	Achieva TX series; Philips Healthcare, Best, the Netherlands	- T1-weighted 3D fast-field echo imaging sequence; TR = 18 ms; TE = 2.4 ms; FOV = 210 × 210 mm^2^, flip angle = 30°; NEX = 1, matrix = 232 × 232; section = 200 - DTI:TR = 8667 ms; TE = 60 ms; FOV = 224 × 224 mm^2^, flip angle = 90° NEX = 1; matrix = 112 × 109; section = 70; section thickness = 2 mm, gap = 2 mm	brain, spinal cord
Wang et al., 2012 [14]	age-matched	Sonata, Siemens, Erlanger, Germany	- 1.5 T - T1-weighted magnetization-prepared rapid acquisition gradient echo sequence - TR: 2070 ms; TE: 3.93 ms; TI: 1110 ms; flip angle: 15°; FOV: 230 mm; slice thickness: 0.9 mm, no gap; matrix: 256 × 256 × 192, NEX: 1	cerebral cortex
Shi et al., 2010 [28]	age- and gender-matched	Sonata, Siemens, Erlangen, Germany	- 1.5T - T2-weighted 3D constructive interference steady state sequence - TR: 11.94 ms; TE: 5.97 ms, flip angle: 70°; FOV: 130 mm; slice thickness: 1 mm; no gap; matrix: 320 × 288; NEX: 1	vestibular system
Domenech et al., 2011 [15]	age-matched	Philips Intera, Best, The Netherlands	- 1.5T - echo planar imaging T2-weighted sequence - TR: 59 ms; TE: 40 ms; flip angle: 50°; voxel size: 1.72 × 1.72 × 5.00; no gap; 12 slices	motor cortical network
Shi et al., 2009 [24]	age- and gender-matched	Sonata, Siemens, Erlangen, Germany	- 1.5 T - magnetization-prepared rapid acquisition gradient echo sequence - TR: 2070 ms; TE: 3.93 ms; TI: 1110 ms; flip angle: 15°; FOV: 230 mm; section thickness: 0.9 mm; no gap matrix: 256 × 256 × 192; NEX: 1	white matter, gray matter, and cerebrospinal fluid
Liu et al., 2008 [13]	age- and gender-matched	Sonata, Siemens, Erlanger, Germany	- 1.5T - rapid acquisition gradient echo sequence - TR = 2070 ms; TE = 3.93 ms; TI = 1110 ms; flip angle = 15 degrees; FOV = 230 mm; slice thickness = 0.9 mm; no gap; matrix = 256 × 256 × 128; number of excitation = 1 - sequence of high-quality isotropic images 0.9 × 0.9 × 0.9 mm - scanning time of each subject 8.5 min	99 anatomical regions of brain
Sun et al., 2007 [20]	age-matched	Gyroscan Intera, Philips Medical Systems, Best, the Netherlands	- 1.5T - turbo spin-echo sequence - TR: 400 ms; TE: 20 ns; slice thickness: 3 mm; gap: 2.5 mm	brain, brainstem, cerebella, spinal cord, the posterior fossa, foramen magnum, and cervical spine
Chu et al., 2007 [19]	age- and gender-matched	Sonata, Siemens, Erlanger, Germany	- 1.5T - magnetization-prepared rapid acquisition gradient echo sequence - TR: 2070 ms; TE:3.93 ms; TI: 1110 ms; flip angle: 15°; FOV: 230 mm; slice thickness: 0.9 mm, no gap, resolution 256 × 256	cerebellar tonsil, foramen magnum, cerebrospinal fluid
Cheng et al., 1999 [10]	age-matched	Gyroscan ASC NT; Philips Medical System, Best, the Netherlands	- 1.5T - turbo spin-echo T1- and T2-weighted sequence - TR, 3.5 s; TE, 120 msec; field of view, 25–40 cm; 256 3 256 matrix; 4 mm slice thickness	the whole spine

**Table 4:** Assessment of risk of bias in included studies using the Newcastle-Ottawa Scale for case–control studies.

Article, Year	Selection				Comparability	Exposure			
	(1) Case Definition is Adequate	(2) Representativeness of the Cases	(3) Selection of Controls	(4) Definition of Controls	(5) Comparability of Cases and Controls	(6) Ascertainment of Exposure	(7) Same Method of Ascertainment for Cases and Controls	(8) Non-Response Rate	(9) Total Score
Noriega et al., 2022 [22]	★	—	★	★	★	—	★	—	5
Carry et al., 2020 [27]	★	★	—	—	★	★	—	—	4
Xue et al., 2018 [23]	★	★	★	★	★	★	★	—	7
Chau et al., 2016 [29]	★	★	★	★	★★	★	★	—	8
Hitier et al., 2015 [26]	★	—	★	★	★	★	★	—	6
Lee et al., 2015 [21]	★	★	★	★	★	★★	★	—	8
Kong et al., 2014 [25]	★	★	★	★	★★	★★	—	—	8
Wang et al., 2012 [14]	★	—	★	★	★	★	★	—	6
Shi et al., 2010 [28]	★	★	—	—	★	★	—	—	4
Domenech et al., 2011 [15]	★	—	★	★	★	★	★	—	6
Shi et al., 2009 [24]	★	★	★	—	—	★	—	—	4
Liu et al., 2008 [13]	★	—	★	★	★	★	—	—	5
Sun et al., 2007 [20]	★	★	★	★	★	★	★	—	7
Chu et al., 2007 [19]	★	★	★	★	★	★	★	—	7
Cheng et al., 1999 [10]	★	★	★	★	★	—	★	—	6

**★**: Items that fulfilled the criteria of minimal bias risk are assigned stars.

## Data Availability

This study did not generate any new datasets. All data analyzed are from publicly available sources, as cited in the manuscript.

## Supplementary Materials

The following supporting information can be downloaded at: https://www.mdpi.com/article/10.3390/children13040499/s1, PRISMA_2020_checklist and Search strategy.

## Abbreviations

The following abbreviations are used in this manuscript:

AIS	Adolescent idiopathic scoliosis
MRI	Magnetic resonance imaging
SEPs	Somatosensory-evoked potentials
DTI	Diffusion tensor imaging
NOS	Newcastle–Ottawa Scale
