# Supplementary material for: Neuromorphological Alterations in the Somatosensory System of Adolescent Idiopathic Scoliosis: A Systematic Review of Magnetic Resonance Imaging Studies
Source: Children (Basel). 2026 Apr 1;13(4):499. doi: 10.3390/children13040499 (PMC13115505; doi:10.3390/children13040499)
Supplement: Supplementary file 1 [file children-13-00499-s001.zip › children-4148810-supplementary/children-4148810-supplementary -new/children-4148810-supplementary.pdf]

Pubmed

#1 “Prognosis”[MeSH Terms] OR “Prognosis”[Title/Abstract] OR “Prognostic*”[Title/Abstract] OR “Epidemiology”[MeSH Terms] OR “Epidemiologic Factors”[MeSH Terms] OR “Epidemiology”[Title/Abstract] OR “Epidemiologic*”[Title/Abstract] OR “Risk*”[Title/Abstract] OR “Predict*”[Title/Abstract] OR “Prevention”[Title/Abstract] OR “Etiology”[Title/Abstract] OR “Etiological”[Title/Abstract] OR “Causality*” [Title/Abstract]

#2 “Observational Study”[Publication Type] OR “Observational”[Title/Abstract] OR “Case-Control”[Title/Abstract] OR “Case Control”[Title/Abstract] OR “Cross-Sectional”[Title/Abstract] OR “Cross Sectional”[Title/Abstract] OR “Cross-Section”[Title/Abstract] OR “Cross Section”[Title/Abstract] OR “Cohort”[Title/Abstract] OR “Perspective”[Title/Abstract] OR “Retrospective”[Title/Abstract] OR “Longitudinal”[Title/Abstract] OR “Comparative”[Title/Abstract] OR “Comparison”[Title/Abstract] OR “Follow-Up”[Title/Abstract] OR “Follow Up”[Title/Abstract] OR “Association”[Title/Abstract] OR “Correlation”[Title/Abstract] OR “Relationship”[Title/Abstract]

#3 #1 OR #2

#4 "Scoliosis"[MeSH Terms] OR "Scoliosis"[Title/Abstract] OR "Scoliotic"[Title/Abstract] OR "Spine Deformit*"[Title/Abstract] OR "Spinal Deformit*"[Title/Abstract] OR " Vertebral Deformit*"[Title/Abstract] OR "Spine Curve*"[Title/Abstract] OR "Spinal Curve*"[Title/Abstract] OR "Vertebral Curve*"[Title/Abstract] OR "Spine Curvature*"[Title/Abstract] OR "Spinal Curvature*"[Title/Abstract] OR "Vertebral Curvature*"[Title/Abstract] OR "Kyphoscoliosis“[Title/Abstract]

#5 "Neuroimaging"[MeSH Terms] OR "neuro-imag*"[Title/Abstract] OR "neuroimage*"[Title/Abstract] OR "magnetic resonance imaging"[Title/Abstract] OR "MRI"[Title/Abstract] OR "cortical thickness"[Title/Abstract] OR "tractography"[Title/Abstract] OR "voxel-based morphometry"[Title/Abstract] OR "VBM"[Title/Abstract] OR "deformation-based morphometry"[Title/Abstract] OR "DBM"[Title/Abstract] OR "cortical thickness"[Title/Abstract] OR "subcortical volumes"[Title/Abstract] OR "Diffusion Weighted Imaging"[Title/Abstract] OR "DWI"[Title/Abstract] OR "Diffusion tensor imaging"[Title/Abstract] OR "DTI"[Title/Abstract] OR "Structural connectivity"[Title/Abstract] OR "surface-based morphometry"[Title/Abstract] OR "SBM"[Title/Abstract] OR "fMRI"[Title/Abstract] OR "functional magnetic resonance imag*"[Title/Abstract] OR "fcMRI"[Title/Abstract] OR "functional connectivity magnetic resonance imag*"[Title/Abstract] OR "Network"[Title/Abstract] OR "Connectivity"[Title/Abstract] OR "iFC"[Title/Abstract] OR "Community Structure"[Title/Abstract] OR "Brain Mapping"[MeSH Terms]

#6 Balance[Title/Abstract] OR Equilibrium[Title/Abstract] OR Postur*[Title/Abstract] OR Motor[Title/Abstract] OR Movement[Title/Abstract] OR Coordination[Title/Abstract] OR Sensor* [Title/Abstract] OR Neuromuscular[Title/Abstract] OR Somatosensory[Title/Abstract] OR Proprioception[Title/Abstract] OR Position Sense[Title/Abstract] OR Kinesthesia[Title/Abstract] OR Vestibular[Title/Abstract] OR Vision[Title/Abstract] OR Visual[Title/Abstract]

#7 #3 AND #4 AND #5 AND #6
